# Bibliographical Department

**Published:** 1881-05

**Authors:** 


### BIBLIOGRAPHICAL DEPARTMENT.

"Nothing extenuate, nor set down aught in malice."

*Lectures in Diseases of the Nervous System, Especially in Women, by L. Weir Mitchell, M. D., Member of the National Academy of Sciences; Physician to the Orthopœdic Hospital and Infirmary for Diseases of the Nervous System; Fellow of the Philadelphia College of Physicians; Member of the New York Academy of Medicine, etc., etc., etc. Philadelphia: Henry C. Lea's Son & Co., 1881.*

We have perused the above volume of 238 pages with pleasure and satisfaction, and feel that its able and justly distinguished author has made in it a most valuable contribution to practical medicine. We are glad to be able to endorse his views most fully in regard to the infrequency of chorea in the negro race. In a long experience as a practitioner on the rice and cotton plantations, and in cities and towns of the South, we have never seen a single case of chorea in the negro race. Six cases in all have come under our observation in a practice of more than a third of a century. Four of those cases were of the caucasian or white race—one adult, and one male child and two female children. The others were a male octoroon (seven-eighths white), and a female quadroon (three-fourths white). The first was nine and the latter seven years old. When preparing a course of lectures on practical medicine, before the war, twenty-one responses from physicians largely engaged in practice on the rice and cotton plantation negroes, upon the subject of chorea and other nervous affections in that race, revealed but a single case of chorea in a full-blooded negro child. In this connection, it may not be inappropriate to state that we have never seen a case of puerperal convulsions in a negress, in several hundred cases of parturition which came under our professional care in that race; whilst mulatto, quadroon and octoroon (admixture of white blood) were not only the subjects of such attacks, but were more frequently so than white women. We heartily commend this unique and well written volume to our professional brethren, and feel assured that those who have not perused its intelligible pages have much in store for their future gratification and use.

*An Introduction to Pathology and Morbid Anatomy: by T. Henry Green, M. D., Lond, Fellow of the Royal College of Physicians; Lond, Physician to Charring Cross Hospital, and Lecturer on Pathology and Morbid Anatomy at Charring Cross Hospital Medical School, etc., etc. Fourth American, from the fifth revised and enlarged English edition, with 138 fine engravings. Philadelphia: Henry C. Lea's Son & Co., 1881. 347 pages. Price $2.25.*

The foregoing is the title-page of a most valuable work in the department of medicine on which it treats. The fourth edition will be found far more interesting and instructive than its predecessors. The wood-cuts are all drawn from microscopical preparations made by Dr. Green, and are true to nature. The text is full and explanatory of the cuts, as well as all the points necessary to be understood by description. We know of no work in our language that could be studied to greater advantage by the student, or one more reliable for reference by the active practitioner.

It is only necessary to add that both the foregoing works are from the excellent and liberal publishing house of H. C. Lea's Son & Co., to assure any one that the type, paper, etc., are all they ought to be.

*The Diseases of Children; a Practical and Systematic Work for Practitioners and Students: by William Henry Day, M. D., Author of Headaches; their Cause, Nature and Treatment; Member of the Royal College of Physicians of London, Physician to the Samaritan Hospital for Women and Children. Second edition. Rewritten and much enlarged. Philadelphia: Prisley Blakiston, No. 1012 Walnut street, 1881. Cloth, $5.00; sheep, $6.00. From Cushing & Bailey, Baltimore.*

A rather careful examination of the above work confirms the good opinion we had formed in advance of its appearance upon our table; and we are prepared to say that it is in harmony with the most modern thought and experience of the profession on the important subject of the diseases of children. It is a thoroughly practical work, and is well worthy of a place in the library of the physician. It is published in the usually excellent style of Mr. Blakiston.

We are also in the receipt—through Messrs. C. & B.—of the following brochures from the same publishers, to wit:

*The Management of Children in Sickness and in Health, a Book for Mothers: by Annie M. Hale, M. D.,*

Should be in the hands of every intelligent mother; and should its precepts be heeded and properly regarded, the loss of life in infancy and childhood would soon be materially lessened.—Price 50 cents.

*How a Person Threatened or Afflicted with Bright's Disease Ought to Live: by Joseph T. Edwards, M. D. Price 75 cents.*

This one conveys valuable rules for the guidance of those for whose use it was written.

*Post-Mortem Examinations, with Special Reference to Medico-Legal Practice, by Professor Rudolph Virchow, of the Berlin Charite Hospital. Translated from the second German edition by Dr. T. P. Smith.*

The name of the author is sufficient guarantee of the value of the book.

The last is one of the *American Health Primers*, entitled *Sea-Air and Sea-Bathing: by John H. Packard, M. D., Surgeon to the Episcopal Hospital.*

During the spring and early summer many of our people will doubtless avail themselves of the excellent facilities for sea-traveling for health or recuperation; and many more of them will find their way to some of the numerous seaside resorts, and all of them would find this little brochure of much value to themselves and families.

Messrs. Cushings & Bailey, of this city, have also placed the following valuable work upon our table, viz.:

*Medical Electricity, a Practical Treatise on the Application of Electricity to Medicine and Surgery: by Roberts Bartholow, A. M., M. D., LL.D., Professor of Materia Medica and General Therapeutics in the Jefferson Medical College of Philadelphia; Fellow of the College of Physicians of Philadelphia; Member of the American Philosophical Society; Honorary Member of the Medical and Chirurgical Faculty of Maryland, etc., etc., etc. With ninety-six illustrations. Philadelphia: Henry C. Lea's Son & Co., 1881.*

At this day, when of book-making there seems to be no end, it is well we can find so many works that are really meritorious and worthy the cost and time required for their perusal. Professor Bartholow's book is one of that class. It is written by an educated general practitioner, and not by a so-called specialist in the practice or application of electricity to disease. It was intended to impart the knowledge to students that is necessary to a practical understanding and intelligent application of one of the most powerful, if not the most potent, agents in nature for the cure of disease, or relief of human suffering.

The same mystery that still envelopes its essential nature, its strange effects upon the contractility of animal tissue, and the metamorphosis it is capable of producing, rendering it a fascinating subject to the scientific physician, afford, also, refuge and shelter to the empiric, as he brandishes his batteries, electrodes and other pharaphernalia appertaining to *his* specialty of electrician, before the credulous or suffering.

Its use should, if possible, be restricted to the intelligent and educated members of the profession, because of its potency for evil, as well as for good. But, unfortunately, this is not possible of accomplishment under "our free system," and our electricians are generally persons who have had but limited practical experience as as physicians, or those of little or no knowledge of the profession, who have adopted the battery as a means of getting their bread. The book is well written, the plates and illustrations good, and the whole make-up of the volume is in the excellent style so characteristic of the well-known publishing house from whence it emanates. The author gracefully dedicates the work to his preceptor, Professor McSherry. It should be in the library of every general practitioner. Price $2.50.

*Domestic Hygiene: a Lecture before the St. Peter's Brotherhood; by F. Donaldson, M. D., Clinical Professor of Throat and Chest, University of Maryland.*

We are indebted to the courtesy of the author for a copy of this admirable discourse, and shall take pleasure in laying certain portions of it before our readers in our "Popular Science Department."

The following journals have been received, and cheerfully placed upon our exchange list, viz.:

*The Sanitary News, the Health Journal of the Mississippi Valley.* Edited and published by R. C. S. and C. A. L. Reid, M. D. Editorial office Hamilton, Ohio.

*The Texas Medical and Surgical Record; a Monthly Journal, Devoted to Medicine and Surgery.* C. H. Wilkinson, M. D., editor. Galveston, Texas, $2,00 per annum.

*The Monthly Review of Medicine and Pharmacy.* Richard V. Mattison, Ph. G., M. D. (University of Pennsylvania). Philadelphia: $1.00 per year.

*Walsh's Retrospect, Washington, D. C.; a Quarterly Compendium of American Medicine and Surgery.* Edited and published by Ralph Walsh, M. D. April, 1881.

This is an excellent periodical and well deserves an extended patronage. It does full work in the department of American medical journalism that it occupies. Its editor, Dr. Walsh, should be supported liberally by his countrymen for the strong stand he has taken in support of our indigenous professional work.

Dr. Samuel Theobald has recently issued the seventh annual report of the Baltimore Charity Eye and Ear Dispensary. 2,260 patients are reported to have been treated within twelve months, at a cost of $241.39.

*The Relation of the Marine Hospital Service of the United States to Commerce, the Public and the Medical Profession. Being*

*Report of the Committee of the Medical Society of the State of California, Showing the Character, Objects and Inutility of Such Services.*

We are indebted to the committee for a copy of the foregoing report, and have read it with surprise and edification. We had never before thought of examining into the matter, but are now fully prepared to endorse the conclusion they have reached—that the Marine Hospital Service is not only not a "charity," but an unmitigated imposition upon the class of people who are ostensibly its beneficiaries. Its operation on the "wards of the Government" is an encouragement to debauch and vice. As we have not space to reproduce the evidence upon which the conclusions of the committee are based, we would earnestly recommend its perusal to all wishing information upon the subject.

We are in the receipt of an interesting lecture by Freeman W. Brophy, M. D., D. D., Lecturer on Dental and Oral Surgery in Rush Medical College, Chicago, entitled, *The Position that Dental and Oral Surgery is Destined to Occupy in America.*

The lecture was made before the New York Odontological Society, February 15th, 1881.

And another valuable paper from the author, Dr. John J. R. Patrick, on *Medical Specialties*, read before the Illinois State Medical Society, thirteenth annual meeting, May 19th, 1880.

Both those pamphlets could be read with pleasure and profit.

